# Molecular Profiling for Predictors of Radiosensitivity in Patients with Breast or Head-and-Neck Cancer

**DOI:** 10.3390/cancers12030753

**Published:** 2020-03-22

**Authors:** Kimi Drobin, Michal Marczyk, Martin Halle, Daniel Danielsson, Anna Papiez, Traimate Sangsuwan, Annika Bendes, Mun-Gwan Hong, Ulrika Qundos, Mats Harms-Ringdahl, Peter Wersäll, Joanna Polanska, Jochen M. Schwenk, Siamak Haghdoost

**Affiliations:** 1 Affinity Proteomics, Science for Life Laboratory, Department of Protein Science, School of Engineering Sciences in Chemistry, Biotechnology and Health, KTH – Royal Institute of Technology, Tomtebodavägen 23, 171 65 Stockholm, Sweden; kimi.drobin@edu.stockholm.se (K.D.); annika.bendes@scilifelab.se (A.B.); mun-gwan.hong@scilifelab.se (M.-G.H.); ulrikaqundos@gmail.com (U.Q.); jochen.schwenk@scilifelab.se (J.M.S.); 2 Yale Cancer Center, Department of Internal Medicine, Yale University School of Medicine, 06511 New Haven, CT, USA; michal.marczyk@yale.edu; 3 Department of Data Science and Engineering, Silesian University of Technology, 44-100 Gliwice, Poland; anna.papiez@polsl.pl (A.P.); Joanna.Polanska@polsl.pl (J.P.); 4 Department of Molecular Medicine and Surgery, Karolinska Institutet, 17176, Stockholm, Sweden; martin.halle@sll.se; 5 Reconstructive Plastic Surgery, Karolinska University Hospital, 17176 Stockholm, Sweden; 6 Department of Clinical Science, Intervention and Technology, Division of ENT Diseases, Karolinska Institutet, 14186 Stockholm, Sweden; daniel@mandibeln.se; 7 Department of Oral and Maxillofacial Surgery, Karolinska University Hospital, 17176, Stockholm, Sweden; 8 Centre for Radiation Protection Research, Department of Molecular Biosciences, The Wenner-Gren Institute Stockholm University, 10691 Stockholm, Sweden; Traimate.Sangsuwan@su.se (T.S.); Mats.Harms-Ringdahl@su.se (M.H.-R.); 9 Department of Radiotherapy, Karolinska University Hospital, 17176 Stockholm, Sweden; peter.wersall@sll.se; 10 University of Caen Normandy, Department of medicine, Cimap-Laria, Advanced Resource Center for HADrontherapy in Europe (ARCHADE), 14076 Caen, France

**Keywords:** radiosensitivity, prediction, plasma proteins, radiotherapy, breast cancer, head-and-neck cancer, skin reaction, biomarker, mandibular osteoradionecrosis, ionizing radiation, radiotherapy side effects, personalized radiotherapy

## Abstract

Nearly half of all cancers are treated with radiotherapy alone or in combination with other treatments, where damage to normal tissues is a limiting factor for the treatment. Radiotherapy-induced adverse health effects, mostly of importance for cancer patients with long-term survival, may appear during or long time after finishing radiotherapy and depend on the patient’s radiosensitivity. Currently, there is no assay available that can reliably predict the individual’s response to radiotherapy. We profiled two study sets from breast (*n* = 29) and head-and-neck cancer patients (*n* = 74) that included radiosensitive patients and matched radioresistant controls.. We studied 55 single nucleotide polymorphisms (SNPs) in 33 genes by DNA genotyping and 130 circulating proteins by affinity-based plasma proteomics. In both study sets, we discovered several plasma proteins with the predictive power to find radiosensitive patients (adjusted *p* < 0.05) and validated the two most predictive proteins (THPO and STIM1) by sandwich immunoassays. By integrating genotypic and proteomic data into an analysis model, it was found that the proteins CHIT1, PDGFB, PNKD, RP2, SERPINC1, SLC4A, STIM1, and THPO, as well as the *VEGFA* gene variant rs69947, predicted radiosensitivity of our breast cancer (AUC = 0.76) and head-and-neck cancer (AUC = 0.89) patients. In conclusion, circulating proteins and a SNP variant of *VEGFA* suggest that processes such as vascular growth capacity, immune response, DNA repair and oxidative stress/hypoxia may be involved in an individual’s risk of experiencing radiation-induced toxicity.

## 1. Introduction

Beside its therapeutic properties against cancer, radiotherapy inevitably involves exposure of normal tissues where late as well as acute adverse effects are dose-limiting factors for the treatment. Numerous attempts have been made to develop an assay that can be used for identifying radiotherapy (RT) patients who will develop severe adverse healthy tissue reactions [1,2,3]. The identification of patients at high risk of developing severe side effects is important as it would allow clinicians to consider a change to the standard available RT protocol and/or combine it with other therapeutic alternatives that reduce the risk of damage to healthy tissues. This is a particularly interesting concept for pediatric cancer patients with long-time survival as well as cancer types where wider surgical margins may be an option to RT, such as breast cancer (BC) and head-and-neck cancer (HNC). The impact of RT on breast reconstructions is another aspect where surgery may be delayed due to the patient’s expected and severe response to RT. However, there is currently no standard test available that can reliably predict radiosensitivity (RS) on an individual level. 

The conformal RT plan is based on 3D scans of the tumor region. Dosimetry errors, leading to overexposure of the healthy surrounding tissue, can usually be ruled out as the cause of severe side effects in a specific organ [4]. Consequently, it can be assumed that a high risk of developing severe side effects is related to the patient´s intrinsic RS. Great efforts have been made to find molecular biomarkers that can be used to identify patients at a high risk of developing side effects [5,6,7,8]. At least six groups of genes involved in cell cycle control, DNA repair, apoptosis, DNA damage signaling, oxidative stress response and inflammatory response, were found to be strongly associated with RS [9,10,11,12]. Promising results have also been published based on the analysis of single nucleotide polymorphisms (SNPs) [5,13,14]. An overlap in the response between the radiosensitive (RS) and radioresistant (RR) study sets has been characteristic of these studies [15]. One of the possible explanations for the observed heterogeneous response is that RS is a multi-mechanistic phenotype [12] and that a combination of assays to test different mechanisms or particular profiles, will be needed for diagnostic purposes.

We have previously investigated the impact of oxidative stress on RS in response to radiotherapy for BC and HNC patients separately. In both study sets, we compared highly radiosensitive patients (RTOG 4) to non-sensitive (RTOG 0) matched controls and were able to find, in ex vivo irradiated blood samples, an association between RS and decreased radiation-induced stress response in terms of serum 8-oxo-dG levels [5,6,16]. In the HNC study set we also found an association between RS documented as mandibular osteoradionecrosis (ORN) after radiotherapy, and a genetic variant of GSTP1 [5]. Given the complexity of the mechanisms responsible for maintaining the integrity of cells, organs and tissues, a single target protein or gene will certainly not be sufficient to diagnose radiotherapy patients with increased RS. It is likely that polymorphisms in DNA gatekeeper and caretaker genes as well as lifestyle factors are involved, and that identification of different types of molecular features and combinations thereof will be needed to develop a diagnostic tool to perform individually tailored radiotherapy. By studying the mechanisms of early and late adverse effects on different types of healthy tissues/organs in response to radiotherapy of different cancer types, it may be possible to reveal if there are mechanisms of fundamental importance for the adverse healthy tissue responses.

Our earlier studies of RS in BC and HNC patients have indicated that the risks involved in developing early and late adverse healthy tissue effects share common mechanisms [5,6,17]. The present study was designed to further develop the mechanistic understanding of individual RS by applying an extended proteomic and genomic approach to analyze blood samples from BC and HNC patients with known RS. The long-term aim is to contribute to the development of diagnostic tools for the identification of patients at risk of extensive side effects before the onset of RT. This work will contribute to a better understanding of the mechanisms causing adverse healthy tissue reactions in response to RT.

## 2. Results

**Figure 1:**
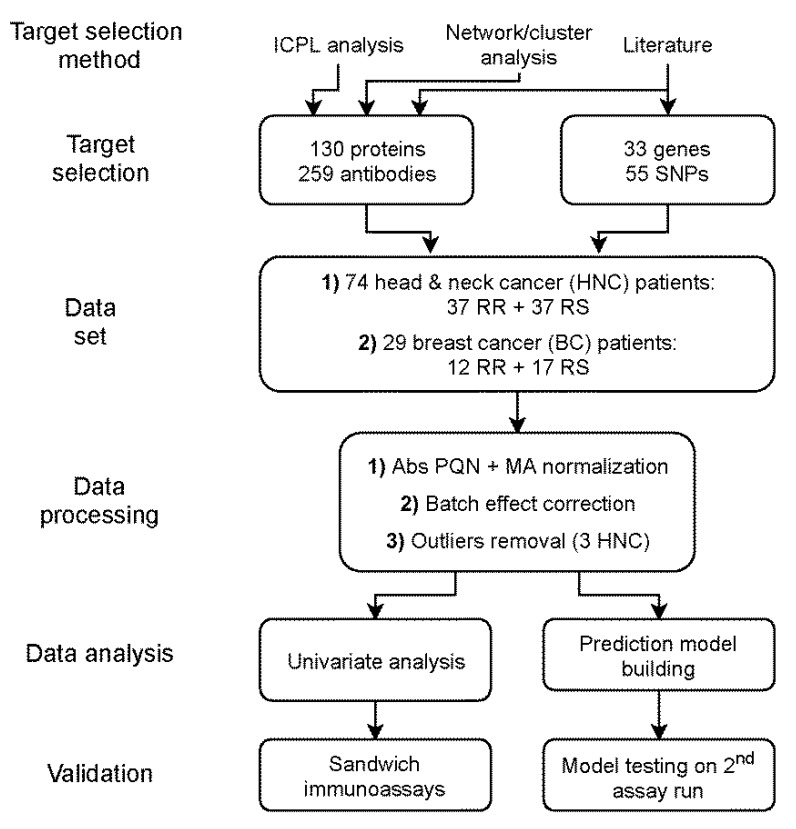
The overall experimental strategy.

An outline of the experimental strategy is shown in Figure 1. The blood samples were drawn from previously radiotherapy-treated breast cancer and head-and-neck-cancer patients with known sensitivity to RT. Plasma was isolated from test tubes with heparin by centrifugation of the tubes. The time point for collecting blood samples was 1 up to 8 years after finishing RT. A list of predicative candidate proteins was generated mainly based on our previous study [6], where intracellular protein profiles from leukocytes of extremely (RTOG 3/4) and normally radiosensitive (RTOG 0/1) breast cancer patients were established using isotope-coded protein labeling (ICPL). Proteins with different abundances in sensitive compared to non-sensitive patients after in vitro exposure to radiation were selected to be included in the present investigation using an antibody bead array approach. In addition, we included other possible protein candidates that were identified using network analysis tools and from literature data of related research on in vitro irradiation of cells [16,18,19,20], as well as proteins associated with DNA repair and signaling. This extended list of targets contained 130 unique proteins for which 1 to 6 antibodies were chosen from the Human Protein Atlas [20]. We applied an exploratory affinity proteomics approach [21] that utilized 259 antibodies (Figure S1A) and assessed data quality and utility using univariate and multivariate statistical methods.

In the BC study set no incomplete or outlying samples were found. In the HNC study set, two samples were excluded prior to the normalization procedure due to technical reasons and one sample was found as an outlier with a different proteomic profile on principal component analysis (PCA) plot (Figure S1b). On the same plot, no significant difference between the two study sets and sample plate (Figure S1c) was found. Finally, data for 100 patients remained and were used for further analysis: 29 were BC patients (12 RR and 17 RS) and 71 were HNC patients (35 RR and 36 RS). There was no imbalance in RS status.

### 2.1. Profiling Patients with Normal or Adverse Healthy Tissue Response to Radiation

**Figure 2:**
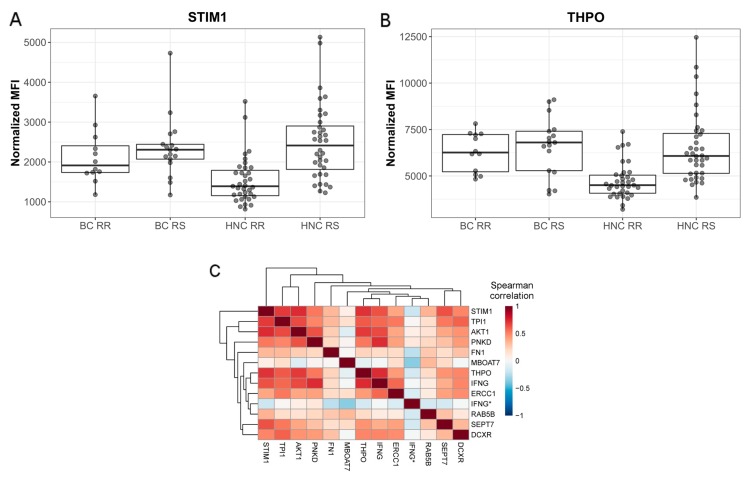
Protein candidates of radiosensitivity measured in exploratory bead arrays. Protein profiles of the top candidates (**A**) STIM1 (**B**) THPO; (**C**) Correlation between significant antibodies identified in univariate analysis.

Using univariate analysis, we found eight protein profiles that were significantly different between RR and RS patients when the Wilcoxon rank-sum test was applied (adjusted *p* < 0.05). Sorted by statistical significance, these were STIM1, THPO, AKT1, IFNG, TPI1, DCXR, PNKD and FN1. The data for STIM1 and THPO are presented in Figure 2a,b, respectively. Application of logistic regression modeling for individual protein profiles resulted in five additional targets, namely SEPT7, ERCC1, IFNG (another antibody), RAB5B and MBOAT7. In all logistic models no correlation between RS status and age, sex or cancer type was found. Detailed results of univariate analysis of all protein profiles are presented in Table S1.

Although important, the univariate analysis does not control the redundancy of the chosen candidate predictors. In case of prediction of the complex response, with a need to cover several alternative pathways, a large set of highly correlated target proteins can be of a smaller predictive power due to the limited number of the independent, informative ones. To reveal the internal structure of the candidates, the relationships between all significant candidates were calculated (Figure 2c) and compared to the distribution of the pairwise correlation coefficients across all proteins being measured (Figure S2). Most of the selected protein profiles were positively correlated, although only a few pairs showed the strong correlation of at least large effect size (r > 0.7, Figure S3d). The highest positive correlation was found between STIM1 and AKT1 (r = 0.73), and IFNG and PNKD (r = 0.73), and the highest negative correlation was found for MBOAT7 and IFNG (r = −0.35, Figure S3b). Annotation of the shortlisted protein using KEGG pathways showed several biological functions related to cancer signaling, immune response, cell proliferation, metabolic pathways and DNA repair.

**Figure 3:**
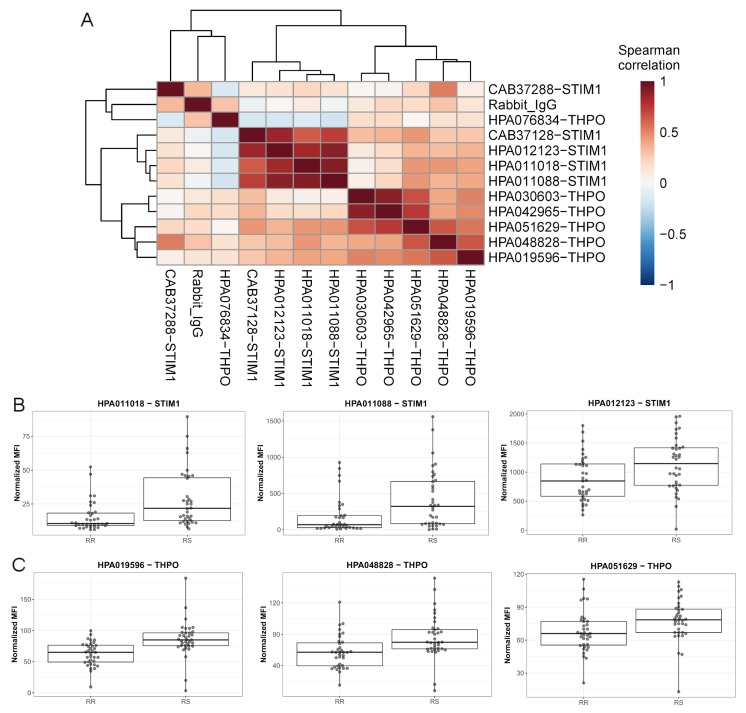
Validation of selected candidate proteins using sandwich immunoassays in the head-and-neck cancer (HNC) data set. (**A**) Correlation between tested antibodies for STIM1 and THPO; Using the sandwich immunoassays to confirm the trends of (**B**) STIM1 and (**C**) THPO in the HNC sample set.

To validate the strongest candidate of RS found in the exploratory bead array assays, we developed dual binder sandwich immunoassays for THPO and STIM1. We used combinations of six antibodies for THPO and five antibodies for STIM1 and a dedicated protocol as described elsewhere [22]. Analyzing the HNC samples, the protein profiles of the different capture antibodies against the same target were highly correlated (Figure 3a and Figure S4). Concordant trends and significant differences were observed for three STIM1 antibodies (HPA011018, HPA011088, HPA012123) and three THPO antibodies (HPA019596, HPA048828, HPA051629), hence confirming our previous observations (Figure 3b,c). 

### 2.2. Patient Genotype and its Association with Radiosensitivity

**Figure 4:**
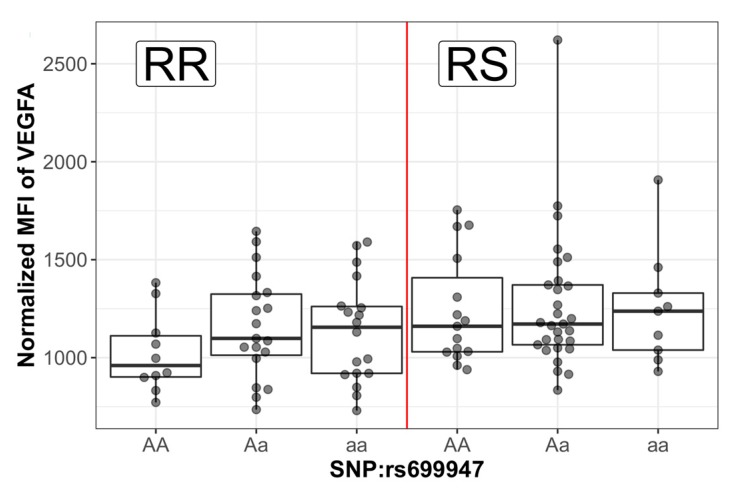
VEGFA protein level for patients with different genotype (AA-wild type, Aa-heterozygous, aa-mutant) grouped by resistance status (RR or RS).

In addition to the analyses of plasma proteins, we analyzed 55 SNPs in both study sets by DNA genotyping of 31 genes reported in the literature to be involved in RS [23,24,25,26,27,28,29]. Among these, we found corresponding protein profiles for 8 genes of which only VEGFA was found significant in univariate analysis of the proteins, but without correction for multiple testing (Table S3). Five out of 55 genotyped SNPs were located in the *VEGFA* gene region. Six additional SNPs were selected for statistical testing by searching for *VEGFA* transcription factors (TFs) using TRRUST v2 database [30]. We found two TFs, for which we had genotyped SNPs: *HIF1A* with five SNPs in our data and *TP53* with one SNP in our data. In total, 11 SNPs of three genes were analyzed. Detailed results of SNP analysis are presented in Table S2. In Figure 4 we show diversity in the protein level of VEGFA for patients with different genotypes of rs699947 SNP grouped by RS status. Only this SNP had significantly different allele frequency in RR and RS patients (*p* = 0.048, without correction for multiple testing). At the genotype level, there were no statistically significant SNPs that could predict RS (Table S3). 

### 2.3. Multiple Protein Model Predictive of Radiosensitivity

**Figure 5:**
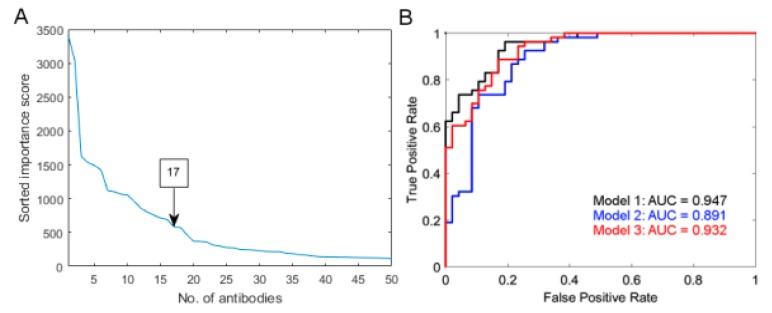
(**A**) Sorted importance score from multiple random validation-based feature selection. Seventeen antibodies were selected based on the “knee” method. (**B**) Receiver-operator curve for three logistic regression models with different numbers and types of features.

Applying the multiple random validation (MRV) approach allowed us to rank antibodies by their correlation with RS status with the use of importance scores (please check Methods). The “knee” method was used on sorted importance scores to select the most relevant antibodies, resulting in an RS signature with 17 antibodies (Figure 5a). Random splitting of the data in the MRV procedure and relatively high number of antibodies in the RS signature could potentially allow selection of proteins that represent more than one biological mechanism responsible for patient RS. Among all proteins selected by importance score, there were five that were significant in the univariate analysis, namely AKT1, FN1, PNKD, STIM1 and THPO. Among others, there were the following proteins: BLVRB, CHIT1, DBNL, FGA (2 antibodies), GCA, PDGFB, PGR, PPARA, RP2, SERPINC1 and SLC4A1. 

First, the prediction model was constructed using all 17 shortlisted profiles (model 1; R^2^ = 0.62; *p* = 2.21 × 10^−10^). Then, the model was reduced to eight proteins giving slightly weaker fit to the data (model 2; R^2^ = 0.49; *p* = 1.68 × 10^−9^). The last model was created by adding one significant SNP, rs699947, to model 2 (model 3; R^2^ = 0.57; *p* = 2.47 × 10^−12^). 

By analyzing the receiver operating characteristic (ROC) curves (Figure 5b) we determined that overall Model 1 had the highest classification performance, while Model 2 had the lowest. Validation of the prediction models on data from the second run of the assay gave a minor decrease in accuracy by 3–4%. This proves the high reproducibility of the assay for selected antibodies and the high classification accuracy of the prediction models. Detailed results of model building and performance assessment are presented in File S1 and the functional association between Model 3 proteins are shown in supportive Figure S5.

In Table 1 we present the relationship of predictors of RS measured by odds ratio (OR). For most of the protein profiles included in the models, an increase in the protein level led to higher odds of patients as RS.

When comparing models, we observe in Model 3 the highest proportion of significant predictor variables. In Table 2, we summarize the performance of classifiers made on logistic regression (LR) models by thresholding LR model output. In the first assay, which was used to construct a model, a misclassification rate of 15% on average was obtained. For Model 1, we observed similar results for BC and HNC patients. For Models 2 and 3, the prediction of RS status was more accurate for the HNC patients, which was caused by reduced specificity for the BC patient group.

## 3. Discussion

We have previously investigated the relationship between individual oxidative stress response and radiosensitivity, and showed that in BC and HNC patients the individual response to oxidative stress plays a significant role in individual radiosensitivity [3,5,17]. There, we applied a proteomic approach to blood samples from a BC study set, showing 40 candidate proteins that were differentially regulated in the sensitive group, a substantial fraction of which were proteins regulating oxidative stress responses [6]. These data showed that the intrinsic cellular oxidative stress response differed between normal responders and patients with severe side effects. Our general conclusion was that the intrinsic regulatory ability to respond to oxidative stress, as well as to the additional stress induced by ionizing radiation (IR), can be an important general factor influencing the sensitivity of cancer and normal tissues to RT. Interestingly, it has been shown by the researchers involved in the Pan-Cancer Analysis Group, that despite the differences between cancers, considering initiation factors, response to the treatments and genomic alterations, there are significant similarities in the genetic and molecular pathways of different cancers that influence the outcome of treatments [31,32,33]. Given the complexity of the mechanisms responsible for maintaining the integrity of the cell, the organ and the tissue, and their response to irradiation of different qualities, a single endpoint cannot be a sufficient RS marker [12,34]. It is likely that combinations of different mechanisms, including polymorphisms in DNA gatekeeper and caretaker genes, as well as lifestyle factors are involved. 

In the presented study, we used a combined genotyping and plasma proteomics strategy to investigate SNPs and circulating proteins from a radiosensitive study set compared to matched radioresistant controls. Proteomic analysis showed similar results in both BC and HNC patients for CHIT1, PDGFB, RP2, SERPINC1, SLC4A, STIM1 and THPO, which were seen as significant positive predictors for RS. The *VEGFA* gene variant rs69947 was retrieved as the only significant SNP in univariate analysis among 55 SNPs measured in both study sets. In a merged genotype and proteomic analysis model, the *VEGFA* SNP and the eight circulating proteins were observed as positive predictors for RS. 

We find the link between the *VEGFA* genotype and the PDGFB protein particularly interesting since they both belong to the same growth factor family and play a significant role in blood vessel formation during hypoxia. The findings support the notion that individual variations in handling radiation-induced tissue hypoxia and vascularization may play a pivotal role in radiation toxicity. Post-irradiation hypoxia is generally accepted as being a trigger of radiation-induced inflammation, angiogenesis and fibrosis [35,36]. Hypoxia also generates reactive oxygen species, increases oxidative stress levels, and mediates the production of inflammatory and fibrogenic cytokines, leading to an increase in vascular permeability and collagen formation together with inducing *VEGFA* gene expression [37]. During the late phase of radiation injury, *VEGFA* up-regulation has been detected in different types of tissues where fibrosis may lead to even further increases in *VEGFA* expression [38,39]. VEGFA is a key determinant of vascular permeability where changes lead to a leakage of plasma proteins preceding collagen deposition, as seen in the late radiation response phase [40,41]. This has further been associated with interstitial hypertension and reduced blood flow, both features that contribute to hypoxic and acidic microenvironments that perpetuate a non-healing tissue response causing a secondary cycle of damage [42,43]. Some genetic variants in the *VEGFA* gene may influence the risk of high-grade late rectal toxicity after radiotherapy for prostate cancer [25]. We have previously seen an increase in both hypoxia and *VEGFA* gene expression patterns in both radiated adipose and vascular tissue from BC and HNC patients, respectively [44,45], but have never studied genetic variants related to this biology. 

In the present study, the plasma levels of THPO and STIM are elevated in BC and HNC RS data sets indicating positive predictors for RS. THPO and STIM1 are two interesting multifunctional proteins, both involved in the regulation of skeletal homeostasis [46,47]. THPO promotes megakaryocytic differentiation, regulates self-renewal activity and the pluripotency of hematopoietic stem/progenitor cells [48] and plays a role in non-homologous end joining DNA repair (NHEJ) [49]. NHEJ is the major DNA double strand breaks (DSBs) repair pathway in mammalian cells and defects in NHEJ proteins, e.g., artemis or XLF, confer marked radiosensitivity since RT induces DSBs [50]. STIM1 has an important role in oxidative stress as well as in hypoxia [51,52], two biological pathways underlying RS. 

CHIT1 and SERPINC1 play roles in the inflammatory response. Human chitinases, such as CHIT1, are playing important roles in innate immune response [53,54,55]. CHIT1 primarily protects the body against chitin-containing pathogens. CHIT1 is expressed by macrophages and neutrophils in response to various pro-inflammatory signals [53,56]. Elevations of CHIT1 activity or plasma levels have been reported in patients with elevated inflammatory response, e.g., Gaucher disease [57], sarcoidosis [58] and Alzheimer’s disease [59]. SERPINC1 is an anti-thrombin protein and has an anti-inflammatory function [60]. Elevated plasma CHIT1 and SERPINC1 in the RS study sets are therefore indicative of elevated inflammatory response. In parallel, elevated SLC4A1 (AE1) and PR2 in RS data sets indicate elevated oxidative stress. SLC4A1 (AE1) belongs to anion exchanger family which is involved in the regulation of intracellular pH [61] and is probably involved in response to oxidative stress [56]. There are presently no data available on the role of RP2 in radiosensitivity. However, a relation between hypoxia, oxidative stress and elevated PR2 has been described in the literature [62,63]. 

The clinical impact regarding the identification of radiosensitive subjects cannot be overestimated in a steadily increasing population of cancer survivors. This is particularly relevant for the studied groups of BC and HNC, where wider surgical margins may be an option to radiotherapy in certain cases. The impact of radiotherapy on both breast and head-and-neck reconstructions is another aspect, where the sequence of surgery and radiotherapy may be altered if the individual response to radiotherapy is expected to be severe and further increase the risk of radiotherapy related surgical complications [64,65]. If the individual radiation sensitivity could be diagnosed before the start of radiation therapy, the radiation therapy could be personalized optimizing the tumor control in normo-sensitive patients and limiting the adverse effects in the group of the most sensitive patients.

Limitations of the study need to be acknowledged. Although we merged study sets of both BC and HNC patients, the generalizability to other cancer types needs to be confirmed. One can ask if HNC cancer patients with osteonecrosis have an active necrotic process in their body. The results could be biased due to this process. To address the question, we collected the blood samples weeks after removal of necrotic ORN by surgery and we included several proteins such as fibronectin and TNF alpha to investigate if any of the patients had an active necrotic process [61]. The study size, limited phenotypic data and blood samples that have been collected up to six years after finishing RT are indeed other limitations. Theoretically, biomarkers of RS could be constantly expressed and/or expressed only after irradiation. As our results show that RS is multifactorial, it could be a combination of the two. Thus bio-sampling before and after exposure would optimize the chance to identify unique biomarkers. However, this can be tested and verified by analyzing prospective data sets where samples before and after treatments are available. Within a newly supported Euratom project, HARMONIC (www.isglobal.org/en/-/harmonic), such an approach has been initiated with the aim of identifying the biomarkers and mechanisms behind RS. Considering the preselected SNP analysis, the increasing availability and decreasing costs of large panel next-generation-sequencing will help us to enhance genotyping beyond 55 pre-selected functional SNPs and probably improve the RS profile in the next step of the project.

CConcerns about the study size and limited phenotypic data were in part overcome by combining two study sets, BC and HNC, and given the fact that the study sets consist of carefully selected and matched radiosensitive and non-sensitive patients. We strongly believe that this comparative study can provide new perspectives on the existing knowledge and bring forward new capabilities to diagnose severe radiosensitivity before RT. The use of a combined experimental approach, together with the availability of sufficiently many and well characterized sets of highly sensitive patients might be important for the identification of underlying mechanisms.

## 4. Materials and Methods

### 4.1. Study Population

This retrospective study was conducted with permission from the Stockholm Regional Ethical Committee, reference number Dnrs 03-621, 06/1413-32, 09-129-32, according to the Declaration of Helsinki. All the donors who participated had given their approval consent.

#### 4.1.1. Head-and-Neck Cancer Study Set

The characteristics of the head-and-neck study set (HNC) have been described previously [5]. During the period 2008 to 2010, 64 patients with osteoradionecrosis (ORN), RS group, that were admitted to the Karolinska hospital were asked to participate in the study. Thirty-seven of them accepted. Thirty-seven matched HNC patients (controls), RR group, were recruited from a database of the Stockholm Regional Cancer Center. The controls were matched for tumor site, irradiation dose to the mandible, standard TNM-classification of malignant tumor and sex. The inclusion criterion was the absence of ORN over a time period between RT and recruitment equal or longer than for the matched case. ORN stage was classified according to Schwartz & Kagan [66]. The patients were given a median tumor dose of 68 Gy delivered in 2 Gy fractions, 5 times per week. The average age of HCN RS as well as RR groups was 64 years. Data on dental extraction, brachytherapy, sex and chemotherapy are available and were included in our previous publication [5]. Based on the 3-dimensional dose planning system, patients who received more than 5% above the total dose to mandible were excluded from the study. We also checked if dental extraction could be the cause of ORN in this study set and found no correlation between ORN and dental extraction [5]. Blood was collected by venipuncture in heparin tubes and kept on ice. The tubes were centrifuged in cold at 300× *g* for 15 min, and plasma was collected and stored at −20 °C until analysis.

#### 4.1.2. Breast Cancer Study Set

As previously described [17], a cohort of 2914 breast cancer patients treated with radiotherapy, including photographs of healthy tissue skin reaction of each patient and an assessment of the sensitivity according to the radiotherapy oncology group (RTOG), was established at Karolinska University Hospital, Stockholm, Sweden. From this cohort, 12 non-sensitive patients (RTOG 0) and 17 sensitive patients (RTOG 4, very rare, <0.03) were selected. The average age for the RTOG 0 group was 52 years and for the RTOG 4 group 51 years. Five patients in the RTOG 0 group and 6 in the RTOG 4 group received chemotherapy prior to the radiation therapy. The patients were treated for their tumors 1–6 years before participation in this study. Blood was collected by venipuncture in heparin tubes and kept on ice. The tubes were centrifuged in cold at 300× *g* for 15 min, plasma was collected and stored at −20 °C until analysis.

### 4.2. Antibody Bead Array Assay

#### 4.2.1. Antibody Selection and Bead Coupling

Antibodies against proteins analyzed in this study were selected from the antibody repository of the Human Protein Atlas (HPA) project. A total of 259 antibodies against 130 unique proteins were selected based on concentration (>0.05 mg/mL) and binding specificity. Each antibody was coupled to a specific population of magnetic, color-coded beads (MagPlex, Luminex corp. Austin, TX, US). Bead identities coupled to rabbit anti-albumin antibodies (Dako, Abcam, Cambridge, United Kingdom) and donkey anti-human IgG antibodies (Jackson Immuno Research Laboratories Inc., Philadelphia, PA, USA) were added as positive controls. IgG from non-immunized rabbits and a bead population processed without antibodies were added as negative controls. The beads were processed, assessed and suspension bead arrays (SBAs) were generated as previously described [67].

#### 4.2.2. Sample Randomization and Bead Array Processing

A 25 µL aliquot of the samples from sensitive and non-sensitive breast cancer and head-and-neck cancer patients were randomized in 96-well microtiter plates prior to analysis using a liquid handling device (Freedom Evo150, Tecan, Männedorf, Switzerland). This was done to avoid bias caused by sequential instrument read-out [68]. Each plate was designed to have a similar distribution of samples concerning age and gender. Samples from the same patient were generally assigned to the same plate. In each 96-well plate, three wells were reserved for a replicated sample pool and at least three wells were kept sample-free to serve as technical controls. Three µL of the samples were then diluted 1:10 in Phosphate Buffer Saline (PBS) (Medicago, Thermo Fisher Scientific, Waltham, MA, USA) and NHS-PEO4-biotin (Pierce, ThermoFisher Scientific, Waltham, MA, USA) was used for labelling [21]. For the transfer of small volumes (<5 µL), a suitable liquid handling device (CyBi-SELMA, CyBio, Analytik Jena, Jena, Germany) was used. The labelled samples were diluted and incubated over-night at room temperature together with the SBA [21]. The beads were washed with 1xPBS 1% Tween20 using a plate washer (EL406, BioTek, Winooski, VT, USA) and an instrument containing a flow cytometer (Flexmap3D, Luminex Corp. Austin, TX, USA) was used for the fluorescence read-out. The assay was performed twice on different days to ensure the robustness and reproducibility of the procedure (1st and 2nd run).

#### 4.2.3. Antibody Validation

To evaluate the selectivity of the antibodies targeting the two proteins STIM1 and THPO, a duplex sandwich immunoassay was built, as previously described [22]. To find matching antibody pairs for the assay, 3 antibodies from the Human Protein Atlas project (HPA011018, HPA011088, HPA012123) and 2 commercial antibodies (S6197 and S6072, Sigma-Aldrich, St. Louis, MO, USA) targeting STIM1, and 6 HPA antibodies (HPA019596, HPA030603, HPA042965, HPA048828, HPA051629, HPA076834) targeting THPO were coupled to a distinct bead population and were combined to form SBAs. The same antibodies were biotinylated as previously described [69], and along with one already biotinylated commercial detection antibody targeting THPO (BAF288, LOT: AKZ0415061, R&D, Systems, Bio-techne, Minneapolis, MN, USA), they were evaluated using a dilution series of plasma samples to assess the performance and duplex capacity. The built sandwich duplex-immunoassay was later conducted on 71 of the HNC patient samples. The samples were diluted 1:10 in an assay buffer consisting of 1x PBS with 0.5% (w/v) polyvinyl alcohol (P8136, Sigma-Aldrich, St. Louis, MO, USA) 0.8% (w/v) polyvinylpyrrolidone (PVP360, Sigma-Aldrich, St. Louis, MO, USA), 0.1% casein (C5890, Sigma-Aldrich, St. Louis, MO, USA) and supplemented with 0.5 mg/mL rabbit IgG (Bethyl Laboratories, Montgomery, TX, US), and incubated with the capture antibody SBA over-night on a shaker and at room temperature. The beads were then washed with 1xPBS 1% Tween20 using a plate washer (EL406, BioTek, Winooski, VT, USA), followed by incubation for 1.5 h with 25 µL of 1 µg/mL detection antibody. The beads were subsequently washed, and 50 µL of 0.5 µg/mL R-phycoerythrin-labeled streptavidin (Invitrogen, ThermoFisher Scientific, Waltham, MA, USA) in 1xPBS 1% Tween20 was added and incubated for 20 min. Lastly, the beads were washed and measured in 60 µL 1xPBS 1% Tween20 using a flow cytometer (Flexmap3D, Luminex Corp. Austin, TX, USA). Raw mean fluorescent intensity MFI values were normalized by subtracting negative control signals from bare beads without any antibody.

### 4.3. Single Nucleotide Polymorphism (SNP) Assay

Genotyping was performed for the 55 SNPs in 33 genes. Genomic DNA was extracted from frozen whole blood using the GeneElute kit (Sigma-Aldrich, St. Louis, Missouri, MO, USA) according to the manufacturer’s protocol. The extracted DNA was quantified with a NanoDrop ND-8000 (ThermoFisher Scientific, Waltham, MA, USA). Genotyping was performed at the Mutation Analysis Facility at the Karolinska University Hospital. The selection of SNPs was based on literature studies and covered functional SNPs in the genes involved in the potential mechanisms of sensitivity to RT, e.g., DNA repair, inflammatory response and oxidative stress response. Genomic DNA was extracted from frozen whole blood using the GeneElute kit (Sigma-Aldrich, St. Louis, Missouri, MO, USA) according to the manufacturer’s protocol. The extracted DNA was quantified with a NanoDrop ND-8000 (ThermoFisher Scientific, Waltham, MA, USA). Genotyping was performed at the Mutation Analysis Facility at the Karolinska University Hospital using iPLEX^®^ Gold chemistry and MassARRAY^®^ mass spectrometry system [70] (Sequenom, San Diego, CA, USA). Multiplexed assays were designed using MassARRAY^®^ Assay Design v4.0 Software and Assay Design Suite v1.0 (www.integratedgenetics.com). The PCR amplification was performed in a total volume of 5 µL containing 10 ng genomic DNA, 100 nM of each amplification primer, 0.5 mM dNTP mix, 3.5 mM MgCl2 and 0.5 U HotStarTaq DNA Polymerase in 1.25× Buffer (Qiagen, Hilden, Germany). The reaction was subjected to the following PCR conditions: 95 °C for 15 min, followed by 45 cycles at 94 °C for 20 s, 56 °C for 30 s, 72 °C for 60 s and a final extension at 72 °C for 3 min. The allele-specific single base extension step was performed using the iPLEX^®^ Gold Reagent and chip II kit (Sequenom, San Diego, Ca, USA) according to Sequenom’s recommended conditions in a total volume of 9 µL. Extension primers were added in four different amounts according to their mass; 6.23 pmol, 8.77 pmol, 11.28 pmol and 13.79 pmol, respectively. Extension products were diluted with 16 µL de-ionized autoclaved water before 7–12 nL was spotted onto a 384-element SpectroCHIP II array ((Sequenom, San Diego, Ca, USA)) using Nanodispenser RS1000 ((Sequenom, San Diego, Ca, USA)). The extension products were subsequently analyzed by MALDI-TOF on a MassARRAY^®^ Analyzer Compact mass spectrometer ((Sequenom, San Diego, Ca, USA)). Genotype calls were manually checked by two persons individually using MassARRAY^®^ TYPER v4.0 Software ((Sequenom, San Diego, Ca, USA)). The genotyping was validated using a set of 14 trio families, in total 42 individuals, with genotype data available through the HapMap consortium (HapMap data release #28) for 90% of the SNPs. Concordance analysis with the HapMap data showed concordance rates of 99.7% for all analyzed SNPs. The parent-offspring-compatibility analysis was performed, and no Mendel errors were observed. Additionally, re-genotyping of 33% of the study samples resulted in 100% concordance.

### 4.4. Data Processing and Analysis

#### 4.4.1. Data Pre-Processing

The statistical environment R [71] and Matlab [72] were used for the evaluation, processing and analysis of the data, unless otherwise stated. Technical assessments of the data, such as calculations of coefficients of variation (CV) within each assay and correlations in between the repeated assays were performed on unprocessed data. Prior to data normalization of the SBA data, outlying samples were detected using principal component analysis (PCA) after excluding the sample-free wells from the data set. To minimize the effects of assay plates we applied multi-dimensional MA-normalization [73] on unprocessed MFI values for proteins. The data was subsequently normalized as described in Kato et al. [68] but using probabilistic quotient normalization and by adjusting the processed data for each antibody individually. COMBAT algorithm [74] was used to correct for batch effects between that data sets due to different time points of analysis and blood sampling. Radiosensitivity status was used as an additional covariate in the linear model used in COMBAT.

#### 4.4.2. Univariate Comparisons

For comparisons between RS and RR patient plasma samples, a Wilcoxon rank-sum test was performed to evaluate the ability of each protein profile to separate the groups. Benjamini–Hochberg algorithm was used as a method for multiple testing correction by controlling the false discovery rate (FDR). Additionally for each protein, logistic regression with protein profile, sex and age as predictors and RS status as a model output were fitted. For the functional annotation of significant proteins KEGG [75] pathway collection was used.

Fisher’s exact test was used in allele and genotype frequency analyses of single nucleotide polymorphisms (SNPs). Next, for each SNP, four genetic interaction models (genotype, additive, dominant, recessive) were investigated by fitting univariate logistic regression model. Akaike information criterion was used to select the best genetic interaction model for each SNP. In all tests, the statistical significance threshold was set to adjusted *p* < 0.05.

#### 4.4.3. Prediction Model Building

Prior to model building, antibodies with *p*-values from univariate analysis higher than 0.2 and opposite expression trend in breast and head-and-neck cancer study sets were removed. Next, the most important and robust antibodies were selected using multiple random validation (MRV) approach. In this approach, the data were split 10,000 times into internal training and validation sets with the proportion 75:25. In each iteration logistic regression (LR) model was built on internal training set and RS status prediction was made on internal validation set. Using the results of the prediction of 10,000 LR models, for each protein profile, an importance score was calculated. The importance score is taking into account the predictive power of the particular classifier measured by the area under the curve and the rank of the component in each LR model signature. The optimal number of antibodies in the model (optimal signature) was found on a sorted importance score curve using the “knee” method. The optimal signature model was built using LR (Model 1). Then, the model was reduced in a backward elimination scheme using Bayes factors calculated from Bayesian Information Criterions (Model 2). The last model was found by adding SNPs data to Model 2 (Model 3). Quality of the model fit was assessed by R2 measure and chi-square test by comparing it to the null model. The threshold value for classifying patients as radio-resistant (RR) or radio-sensitive (RS) was found by minimizing weighted prediction error. Performance of the classifiers was measured by area under the ROC curve (AUC), sensitivity, specificity and weighted classification error. Independent data from the second run of the same assay were used to validate the accuracy and reproducibility of all prediction models.

### 4.5. Data Availability

The data is available upon request.

## 5. Conclusions

Our findings demonstrate that the individual vascular growth capacity, inflammation, oxidative stress, DNA repair and tissue hypoxia may influence the risk of radiation toxicity. If confirmed in additional studies and for other cancer types, our findings can contribute to the development of predictive risk models for the occurrence of adverse health effects following radiotherapy. In the best scenario, the model will be valid before the start or RT or within a few days after the start of RT, so it will still be possible to modify the therapy protocol. Increasing knowledge about the underlying biology of individual RS will thus help to establish tailored radiotherapy. This will allow treatment to be better adapted to radiosensitive individuals and thus minimize adverse health effects and ultimately improve the quality of life in a steadily increasing population of cancer survivors.

## Figures and Tables

**Table 1:** Relationship between chosen antibodies and single nucleotide polymorphisms (SNPs), and radiosensitivity status measured by odds ratio with 95% CI calculated in the multiple logistic regression model. The value of odds ratio for antibodies was scaled to represent the impact of a 100-unit increase, instead of a 1-unit increase.

Predictor	Model 1	Model 2	Model 3
HPA011088 - STIM1	1.14 (0.99;1.31)	1.13 (1.01;1.26) *	1.20 (1.07;1.36) *
HPA011325 - PDGFB	4.36 (1.42;13.39) *	2.37 (1.07;5.27) *	3.86 (1.50;9.91) *
HPA010134 - PNKD	1.24 (0.61;2.51)	1.35 (0.86;2.10)	1.38 (0.77;2.48)
HPA030603 - THPO	1.06 (0.97;1.16)	1.05 (0.99;1.12)	1.08 (1.01;1.16) *
HPA010115 - CHIT1	1.19 (1.03;1.38) *	1.08 (0.98;1.19)	1.17 (1.02;1.33) *
HPA000909 - RP2	2.36 (0.65;8.60)	1.46 (0.64;3.34)	2.19 (0.73;6.56)
HPA001816 - SERPINC1	0.93 (0.85;1.01)	0.95 (0.89;1.00)	0.92 (0.86;0.99) *
HPA063911 - SLC4A1	0.74 (0.45;1.24)	0.55 (0.36;0.83) *	0.40 (0.23;0.71) *
HPA004156 - AKT1	2.56 (0.76;8.63)	-	-
HPA035034 – GCA	0.77 (0.56;1.056)	-	-
HPA027066 - FN1	1.06 (0.96;1.17)	-	-
HPA064755 – FGA	0.96 (0.89;1.04)	-	-
HPA051370 – FGA	1.12 (0.99;1.27)	-	-
HPA027735 - DBNL	0.29 (0.08;1.09)	-	-
HPA041937 - BLVRB	1.11 (0.84;1.46)	-	-
HPA004819 – PGR	0.36 (0.16;0.81) *	-	-
HPA051420 - PPARA	0.74 (0.50;1.11)	-	-
Rs69947 – AA/AC	-	-	1
Rs69947 – CC	-	-	0.03 (0.00;0.22) *

* Significant predictor in the model at alpha = 0.05.

**Table 2:** Performance of logistic regression (LR) classifiers on 1st assay run used to build models and 2nd assay run used to test models. Sens is sensitivity, Spec is specificity and WErr is weighted classification error. BC and HNC are the results of classification for all patients, while BC is the results for breast cancer patients and HNC for head-and-neck cancer patients.

Phase	Model	BC+HNC			BC only			HNC only		
		Sens	Spec	WErr	Sens	Spec	WErr	Sens	Spec	WErr
Training	1	**0.96**	0.81	**0.11**	**1.00**	**0.67**	**0.17**	0.94	0.86	0.10
	2	0.92	0.74	0.17	0.82	0.33	0.42	**0.97**	0.89	**0.07**
	3	0.89	**0.83**	0.14	0.82	0.50	0.34	0.92	**0.94**	**0.07**
Testing	1	**0.89**	**0.83**	**0.14**	**0.94**	**0.67**	**0.20**	0.86	0.89	0.13
	2	0.87	0.70	0.21	0.82	0.25	0.46	**0.89**	0.86	0.13
	3	0.85	0.81	0.17	0.76	0.50	0.37	**0.89**	**0.91**	**0.10**

In each column, the best result is highlighted in bold.

## Supplementary Materials

The following are available online at https://www.mdpi.com/2072-6694/12/3/753/s1: Figure S1: Quality control of protein data; Figure S2: The distribution of the pairwise correlation coefficient across all proteins within the set of candidate proteins (predictors) being marked as separate dots below the histogram; Figure S3: Circos plots showing correlation between protein pairs within the set of candidate predictors limited to those predictors being at of least small effect (abs(r) > 0.1 panel A), at least medium (abs(r) > 0.3, panel B), at least large (abs(r) > 0.5, panel C) and very large (and(r) > 0.7, panel D); Figure S4: Protein validation using sandwich immunoassay; Figure S5: String DB association of proteins included in model 3; File S1: Detailed results of prediction model building; Table S1: Univariate analysis results of proteins measured using bead array assay; Table S2: Univariate analysis results of proteins measured using sandwich immunoassay; Table S3: SNP genotyping results.

## Fundings

The work was funded by The Swedish Cancer Society (Cancerfonden) grant no. CAN 2017/494 (S.H.), The Swedish Radiation Safety Authority (S.H.) National Science Center OPUS grant no. UMO-2015/19/B/ST6/01736 (J.P.) and SUT grant for support and development of research potential (A.P.). Support from the Knut and Alice Wallenberg Foundation for the Human Protein Atlas, Science for Life Laboratory for Plasma Profiling Facility, Erling Persson Foundation for the KTH Centre for Precision Medicine, the SRA grant CancerUU from the Swedish Government are also acknowledged (J.M.S.).
