# Supplementary figures and images for: Molecular Profiling for Predictors of Radiosensitivity in Patients with Breast or Head-and-Neck Cancer
Source: Cancers (Basel). 2020 Mar 22;12(3):753. doi: 10.3390/cancers12030753 (PMC7140105; doi:10.3390/cancers12030753)

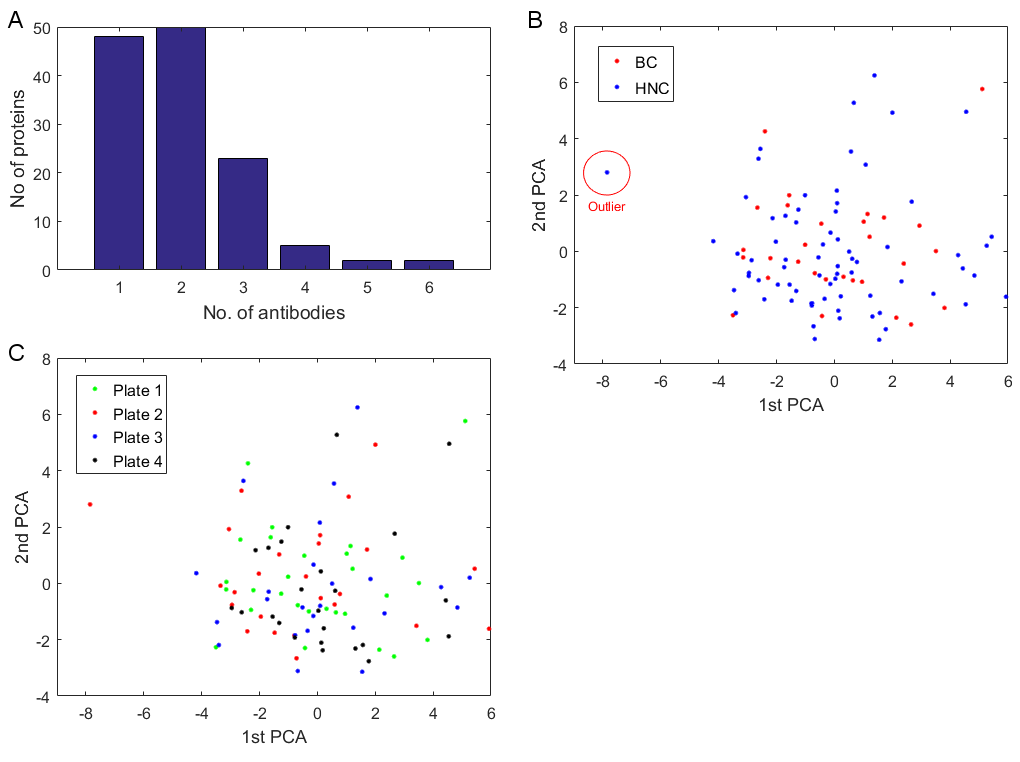

Supplement: Supplementary file 1 [file cancers-12-00753-s001.zip › Supplementary files/FigureS1.tif]

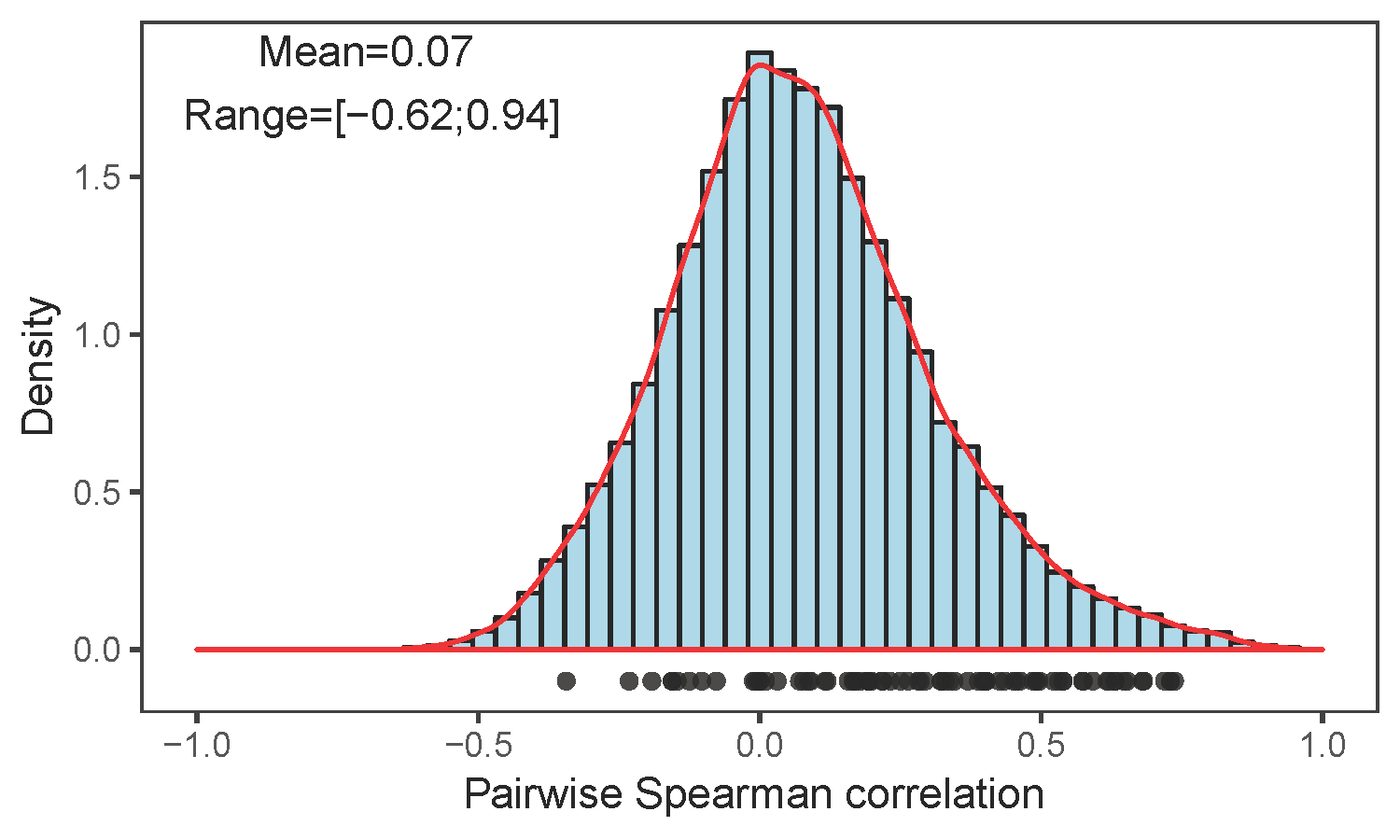

Supplement: Supplementary file 1 [file cancers-12-00753-s001.zip › Supplementary files/Figures2.tif]

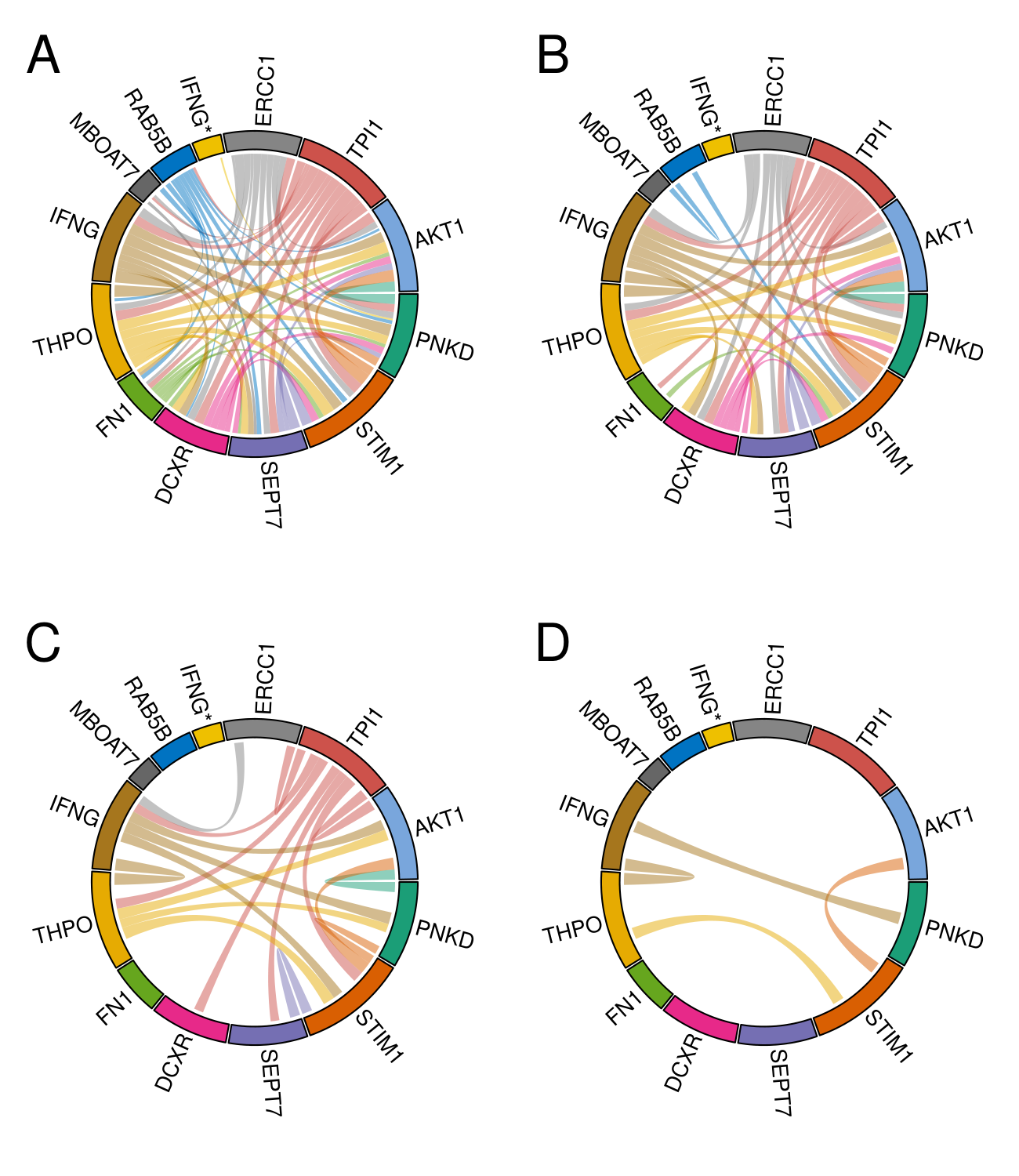

Supplement: Supplementary file 1 [file cancers-12-00753-s001.zip › Supplementary files/FigureS3.tif]

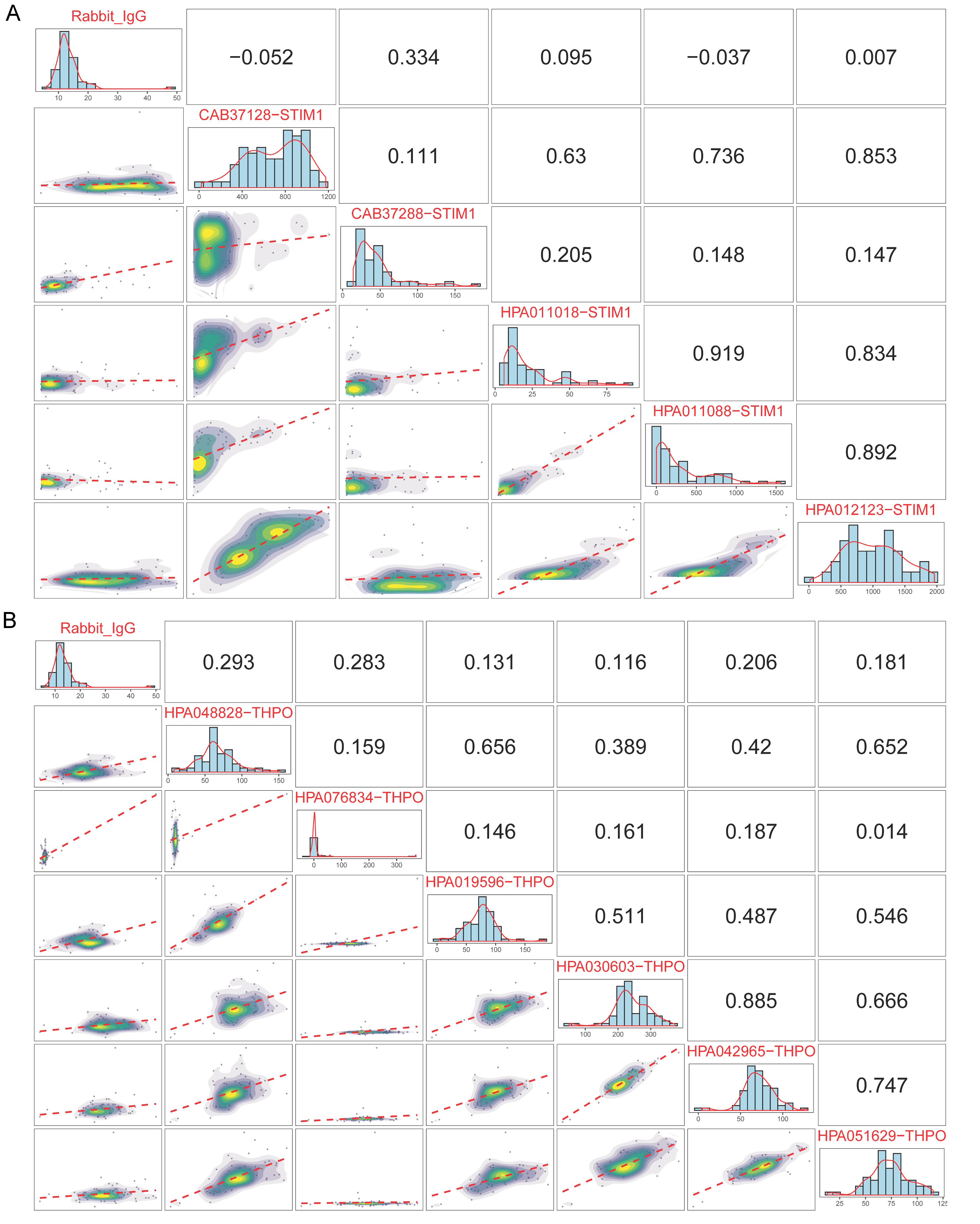

Supplement: Supplementary file 1 [file cancers-12-00753-s001.zip › Supplementary files/FigureS4.tif]

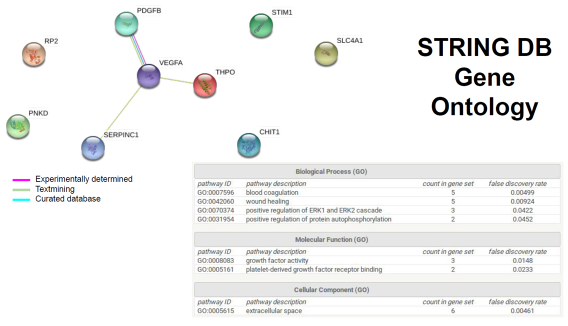

Supplement: Supplementary file 1 [file cancers-12-00753-s001.zip › Supplementary files/FiguresS5.tif]
